# Concentration-dependent effects of fermented spent coffee grounds and contrasting effects of earthworms on growth and phytochemicals in medicinal plant *Glechoma longituba*

**DOI:** 10.1371/journal.pone.0339185

**Published:** 2025-12-17

**Authors:** Bing-Nan Zhao, Zi-Yang Xie, Jia-Ning Liu, Xiao-Ran Chen, Xin-Xin Wang, Jia-Yi Li, Rui Zhang, Chao Si

**Affiliations:** 1 School of Life Science and Engineering, Handan University, Handan, China; 2 College of Life Sciences, Hebei University, Baoding, China; Universidade de Vigo, SPAIN

## Abstract

Fermented spent coffee grounds (FSCG) serve as a valuable soil amendment to improve soil structure and fertility, while earthworms play a well-established role in enhancing soil processes and plant growth. However, their combined effects on bioactive compound accumulation in medicinal plants remain unclear. This study investigated the individual and interactive effects of FSCG (0%, 10%, and 20%, v/v) and earthworms (with and without *Pheretima guillelmi*) on the growth and phytochemical content of *Glechoma longituba*, a common medicinal herb, under greenhouse conditions. Results showed that 10% FSCG generally promoted plant growth, whereas 20% FSCG generally enhanced the accumulation of total flavonoids, chlorogenic acid, and soluble protein. Earthworms enhanced aboveground biomass and node number but significantly reduced chlorogenic acid content. These findings highlight the potential of FSCG as a sustainable soil amendment in medicinal plant cultivation and underscore the need to consider earthworm activity when optimizing both plant biomass and phytochemical quality.

## Introduction

Medicinal plants hold significant therapeutic value and are integral to both traditional Chinese medicine and modern healthcare systems [1,2]. These plants are used across multiple sectors, including natural product extraction, food production, and the chemical industry [3–6]. In recent times, global demand for medicinal plants is rising. However, over-exploitation through human collection has caused a critical shortage of many medicinal resources [7–9]. Artificially cultivated medicinal plants also face challenges such as varietal admixture, degeneration, and low yields [3,8,10]. Therefore, improving both the yield and quality during cultivation is essential to meet medical needs and ensure a stable supply of high-quality products [11,12].

Coffee consumption has surged worldwide in recent years. This generates large amounts of spent coffee grounds (SCG), estimated at 8–15 million tons per year [13–16]. SCG containing high levels of polyphenols, tannins, and caffeine may pose risks to environmental and soil ecosystems, necessitating the development of effective recycling strategies [17–20]. Notably, SCG also contains modest amounts of nitrogen, phosphorus, and potassium [21–23], suggesting its potential as a natural fertilizer or soil conditioner to improve soil structure and provide plant nutrients [24–26]. Nevertheless, the direct incorporation of untreated spent coffee grounds into topsoil beyond a certain concentration has been shown to inhibit the growth of various crops, including broccoli, cress, leek, lettuce, radish, sunflower, and viola [20,24–28]. Fermentation is recommended to reduce these adverse effects [15,29,30]. Fermented spent coffee grounds (FSCG) have a neutralized pH and lower phenolic and tannin content. They also retain nitrogen and minerals, promoting growth in plants like lettuce and tomato [15,29,30]. However, current research on FSCG primarily focuses on common vegetable crops, with its effects on medicinal plants remaining largely unexplored.

Earthworms, as key soil macrofauna, improve plant performance through multiple mechanisms [31–34]. Their burrowing and casting activities enhance soil structure, increase water retention, and promote root development [35–37]. Additionally, earthworms facilitate the decomposition of organic matter via gut-mediated processes, thereby accelerating nutrient cycling and enhancing nutrient bioavailability [38,39]. These soil modifications lead to significant improvements in key plant traits such as germination rate, leaf area, and biomass accumulation [36,40,41]. Moreover, earthworms reshape soil microbial community structure and diversity, which further influences plant physiology and secondary metabolism [42]. They are also capable of surviving and reproducing in organic-rich amendments, such as SCG, thereby continuously modifying soil properties and indirectly influencing plant performance [43–46]. Nevertheless, few studies have examined these interactions in medicinal plants. It remains unclear whether earthworms interact with FSCG to influence medicinal plant growth and secondary metabolite production.

Previous studies show that earthworms can interact synergistically with soil amendments such as biochar [47,48]. They improve its integration into soil and enhance its beneficial effects [47,48]. Similarly, the introduction of earthworms into soils treated with FSCG may enhance nutrient availability and improve soil structure, thereby promoting plant growth. To test the potential interactive effects of FSCG and earthworms on the growth and active constituent accumulation of medicinal plants, we conducted an experiment using *Glechoma longituba* (Lamiaceae), a common clonal medicinal species. We grew plants in soil with 0%, 10%, or 20% (v/v) FSCG, with or without earthworms (*Pheretima guillelmi*). We tested the following hypotheses: (1) The addition of FSCG influences the growth and phytochemical content of *G. longituba* in a rate-dependent manner and (2) earthworms interact with FSCG, thereby modulating its effects on plant growth and phytochemical accumulation in *G. longituba*.

## Materials and methods

### Plant species

*Glechoma longituba* (Nakai) Kuprian. (Lamiaceae) is a perennial clonal herb [49–51]. It is native to Europe and North America, and widely distributed across China, except Qinghai, Gansu, Xinjiang, and Tibet [51–53]. This species has a monopodial stolon with nodes. Each node can produce potential ramets [52,54,55]. *G. longituba* is valued for its medicinal properties, attributed to bioactive constituents such as chlorogenic acid and flavonoids concentrated in its aerial parts [34,51,56–58].

*G. longituba* plants used in this experiment were purchased from a commercial supplier (Shanghai, China). They were acclimatized for several weeks in a greenhouse at Handan University, Handan, Hebei Province, China (36°34′N, 114°29′E) prior to experimental initiation.

### Earthworm species

*Pheretima guillelmi*, an earthworm species of the genus Pheretima, inhabits deep soil layers and primarily feeds on litters [36,59,60]. Adults measure 15−25 cm in length and 5−8 mm in width, with a characteristic green-yellow to gray-blue dorsal pigmentation [36,59]. This species is widely distributed in southern China [61–63]. Recently, it has been utilized for soil quality improvement due to its ability in regulating soil physicochemical properties and microbial community composition [50,62,64]. The earthworms used in this study were obtained from a commercial supplier in Jurong, Jiangsu Province, China. They were acclimatized for two weeks in a plastic container covered with a shade cloth (70% shading rate) inside the same greenhouse where plant materials were acclimatized. During acclimatization, air temperature and humidity in the greenhouse were continuously monitored using a temperature logger (RC-4HC, Elitech, Jingchuang Electric Co., Ltd., China), with recordings taken every two hours. Average readings were 27°C and 63.3%, respectively.

### Experimental design

This experiment utilized a two-factor completely randomized design with interaction. One factor was FSCG concentration at 0%, 10%, and 20% (v/v). These FSCG concentrations were selected to represent a gradient ranging from a beneficial dose to a higher, potentially stress-inducing dose, thereby allowing us to capture the full spectrum of plant responses. Another factor was earthworm presence (with or without). Each treatment combination was replicated five times, a level that effectively balanced statistical power with practical limitations related to plant material availability and spatial constraints, resulting in a total of 30 experimental units.

The SCG were collected from a local coffee shop in Handan, China, and mixed with an organic fertilizer decomposing inoculant (450 g inoculant per m^3^ SCG; Dewoduo Fertilizer Co., Hengshui, China). The mixture underwent aerobic fermentation for 73 days. Fermentation was considered complete when the temperature dropped below ambient levels and remained stable. The base substrate consisted of commercial potting soil (Dewoduo Fertilizer Co., Hengshui, China; pH: 6.0, total organic matter: 439.8 g kg^-1^, total N: 7.9g kg^-1^, total C: 224.7 g kg^-1^, total P: 0.9 g kg^-1^) and sieved (10 mm mesh) topsoil (depth of 0–20 cm; pH: 7.8, total organic matter: 17.1 g kg^-1^, total N: 0.83 g kg^-1^, total C: 20.37 g kg^-1^, total P: 0.8 g kg^-1^) from the Handan University campus (1:1 v/v). FSCG (soluble sugar: 25.0 g kg^-1^, soluble protein: 138.2 g kg^-1^) was incorporated into the base substrate at 0%, 10%, or 20% (v/v) to create treatment soils.

On July 13, 2024, 256 fragments (each consisting of one node and a pair of leaves) of *G. longituba* were excised from stock plants and pre-rooted in seed trays containing a 1:1 (v/v) sand: vermiculite (1–2 mm particle size) mixture. After 11 days of pre-cultivation, 30 uniform fragments exhibiting one initial node, one leaf pair, and two axillary ramets were selected and transplanted individually into the center of pots (20 cm diameter × 15.4 cm height) containing the treatment soils.

On July 25, 2024, half of the pots from each treatment group exposed to different FSCG levels were randomly selected. Three adult earthworms were introduced in each pot, with the number determined according to the density of typical farmland ecosystems (approximately 30 adult individuals per square meter) [65]. On the 20th and 50th days after the initial introduction of earthworms, three earthworms per pot were added to compensate for observed escape and maintain the target population density throughout the experimental period. The experiment was conducted in the same greenhouse at Handan University where the plants were acclimatized, with all pots randomly arranged on a bench in the greenhouse. During the experiment, continuous monitoring using the previously described temperature logger recorded air temperature and humidity in the greenhouse at two-hour intervals, yielding average values of 28°C and 58.9%, respectively. Soil moisture was kept moist by daily watering. The newly produced ramets from each portion were allowed to root in their respective original pots. The experiment concluded on October 4, 2024.

### Measurements and data analysis

At harvest, the soil matrix attached to the plant roots of *G. longituba* was carefully removed, and the node number was counted. Subsequently, the plants were divided into shoots and roots, which were then heated at 105°C for 30 minutes for enzyme deactivation. Subsequently, they were dried at 70°C to constant weight and weighed. Dried samples were stored for determination of total flavonoids and chlorogenic acid.

Total flavonoid content was quantified spectrophotometrically following Liu et al. (2021) [66] with modifications. Chlorogenic acid concentration was determined spectrophotometrically referring to Oteef et al. (2022) [67]. Soluble sugar and soluble protein contents were measured using anthrone and Coomassie brilliant blue methods, respectively [68]. The total biomass was calculated by summing the aboveground and root biomass, and the root-shoot ratio was calculated by dividing the root biomass by the aboveground biomass.

A two-way ANOVA was used to test the effects of FSCG, earthworms, and their interaction on a series of plant measurements about plant growth, morphology and bioactive constituents. Subsequent post-hoc Tukey tests were performed to compare mean differences across FSCG levels within each earthworm treatment. Data transformations were applied where necessary to meet homoscedasticity, with specific methods noted in results tables. Two replicates from the 0% FSCG without earthworm treatment were excluded from all measures and analyses due to plant death that was caused by transplantation stress. All statistical analyses were conducted using SPSS 22.0 (IBM Corp., Armonk, NY, USA).

## Results

### Effects of FSCG and earthworms on growth and morphology

**Fig 1:**
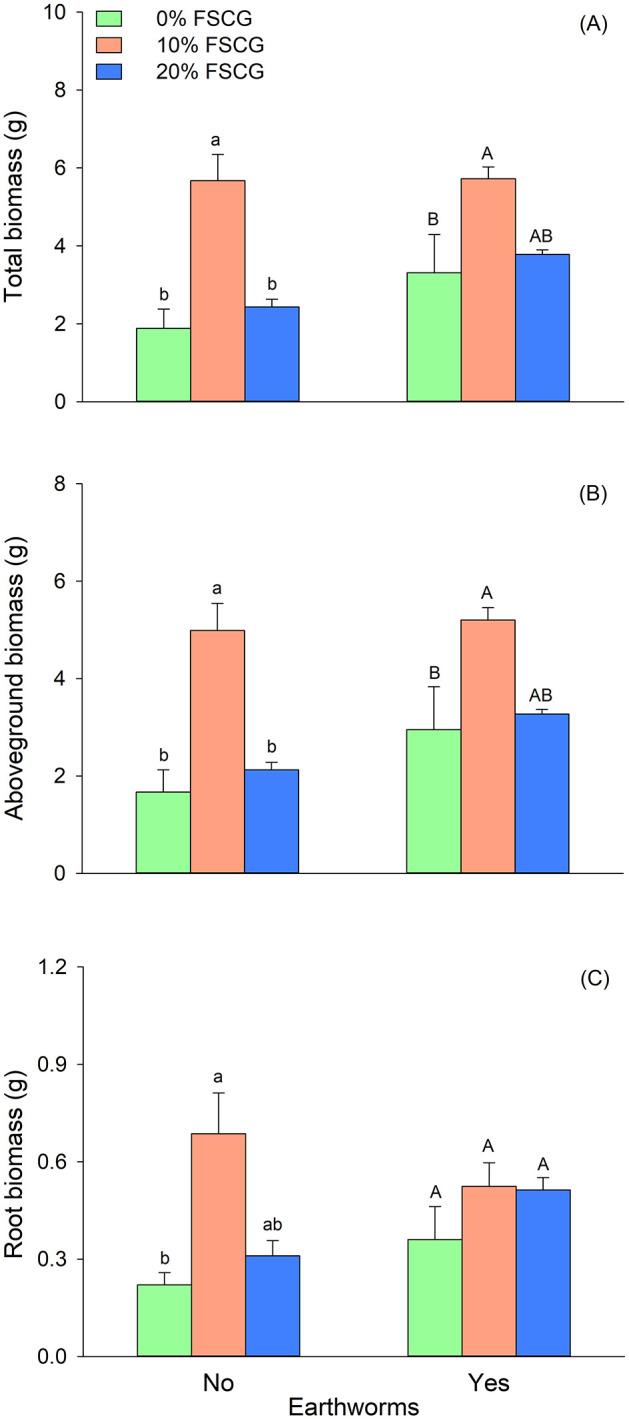
Effects of fermented spent coffee grounds and earthworm on total biomass (A), aboveground biomass (B), and root biomass (C) of *Glechoma longituba.* Bars and vertical lines represent mean and SE. Different lowercase (a, b) and uppercase letters (A, B) denote significant differences among FSCG concentrations in treatments without and with earthworms, respectively (Tukey’s test).

**Fig 2:**
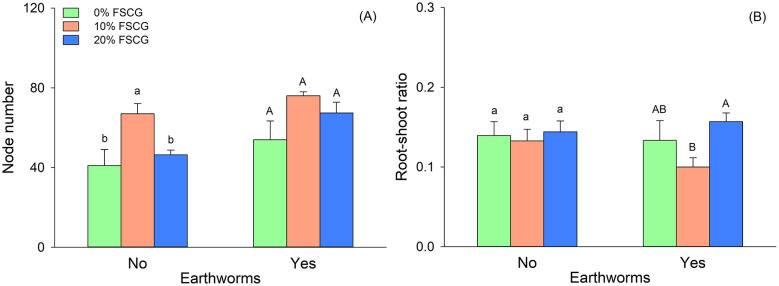
Effects of fermented spent coffee grounds and earthworm on node number (A), and root-shoot ratio (B) of *Glechoma longituba.* Bars and vertical lines represent mean and SE. Different lowercase (a, b) and uppercase letters (A, B) denote significant differences among FSCG concentrations in treatments without and with earthworms, respectively (Tukey’s test).

Two-way ANOVA revealed that FSCG had a significant effect on *G. longituba* biomass (total, aboveground, root) and node number (*P* < 0.05), but not on root-shoot ratio (Table 1). Overall, treatment with 10% FSCG significantly increased biomass and node number. Earthworm presence significantly increased aboveground biomass and node number (*P* < 0.05; Table 1; Fig 1B, 2A), but had no significant effect on total biomass, root biomass, or root-shoot ratio. No significant FSCG × Earthworm interaction was detected for any growth measurements (Table 1).

**Table 1:** Analysis of variance of the effects of fermented spent coffee grounds, earthworm, and their interaction on growth performance of *Glechoma longituba*.

Variable	Fermented spent coffee grounds (FSCG)		Earthworm (E)		FSCG × E	
	F_2, 22_	*P*	F_1, 22_	*P*	F_2, 22_	*P*
Total biomass	**17.5**	**< 0.001**	4.1	0.056	1.0	0.397
Aboveground biomass	**18.8**	**< 0.001**	**4.7**	**0.042**	0.7	0.504
Root biomass^a^	**7.6**	**0.003**	2.0	0.169	2.2	0.139
Node number	**8.2**	**0.002**	**8.8**	**0.007**	0.6	0.564
Root shoot ratio^a^	3.2	0.061	0.9	0.351	1.4	0.259

^a^Natural log transformation.

Degrees of freedom (subscript for “F”), F and *P* values are given. Values are in bold when *P* ＜ 0.05.

Without earthworms, the Tukey HSD test showed that plants grown in 10% FSCG exhibited the highest total biomass, aboveground biomass, and node number (*P* < 0.05; Fig 1A, 1B, 2A). Root biomass was also significantly greater in the 10% FSCG treatment compared to the 0% FSCG treatment (Fig 1C). There was no significant difference in root-shoot ratio between the three FSCG treatments (Fig 2B).

In the presence of earthworms, the Tukey HSD test showed that total biomass and aboveground biomass were significantly higher in the 10% FSCG treatment than in the 0% FSCG treatment (*P* < 0.05; Fig 1A, 1B). Root-shoot ratio was significantly lower in the 10% FSCG treatment than in the 20% FSCG treatment (*P* < 0.05; Fig 2B). While root biomass and node number did not show differences between the three FSCG treatments (Fig 1C, 2A).

### Effects of FSCG and earthworms on phytochemical content

**Fig 3:**
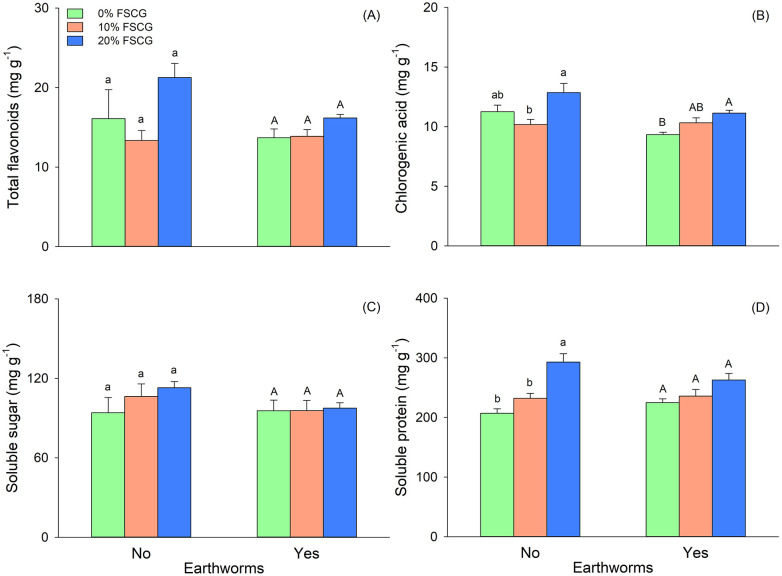
Effects of fermented spent coffee grounds and earthworm on the content of total flavonoids (A), chlorogenic acid (B), soluble sugar (C), and soluble protein (D) of *Glechoma longituba.* Bars and vertical lines represent mean and SE. Different lowercase (a, b) and uppercase letters (A, B) denote significant differences among FSCG concentrations in treatments without and with earthworms, respectively (Tukey’s test).

FSCG significantly influenced total flavonoids, chlorogenic acid, and soluble protein content (*P* < 0.05), but not soluble sugar content (Table 2). Overall, contents of total flavonoids, chlorogenic acid, and soluble protein were higher under 20% FSCG than under other levels (Fig 3A, 3B, 3D). Earthworm presence significantly reduced chlorogenic acid content (*P* < 0.05; Table 2; Fig 3B). No significant FSCG × Earthworm interaction was observed for any phytochemical measurement (Table 2).

**Table 2:** Analysis of variance of the effects of fermented spent coffee grounds, earthworm, and their interaction on content of total flavonoids, chlorogenic acid, soluble sugar, and soluble protein of *Glechoma longituba*.

Variable	Fermented spent coffee grounds (FSCG)		Earthworm (E)		FSCG × E	
	F_2, 22_	*P*	F_1, 22_	*P*	F_2, 22_	*P*
Total flavonoids^b^	**6.8**	**0.005**	2.8	0.107	1.7	0.204
Chlorogenic acid^a^	**9.2**	**0.001**	**8.8**	**0.007**	3.1	0.067
Soluble sugar^a^	1.0	0.389	1.5	0.239	0.5	0.593
Soluble protein^a^	**17.3**	**< 0.001**	< 0.1	0.910	2.4	0.110

^a^Natural log transformation. ^b^Square root transformation.

Degrees of freedom (subscript for “F”), F and *P* values are given. Values are in bold when *P* ＜ 0.05.

Based on the Tukey HSD test results, without earthworms: Chlorogenic acid content was significantly higher under 20% FSCG than under 10% FSCG (*P* < 0.05; Fig 3B). Soluble protein content peaked in the 20% FSCG treatment (Fig 3D). Total flavonoids and soluble sugar did not display significant trend between all FSCG treatments (Fig 3A, 3C). With earthworms: Chlorogenic acid content was significantly elevated under 20% FSCG compared to the 0% FSCG treatment (*P* < 0.05; Fig 3B). Total flavonoids, soluble sugar and soluble protein content did not display significant trend between all FSCG treatments (Fig 3A, 3C, 3D).

## Discussion

### Responses of growth and morphology

Previous studies have showed that the addition of FSCG can improve plant growth while reducing reliance on synthetic fertilizers [29,69]. For example, partially replacing commercial peat with FSCG induced an enhanced plant growth response in basil (*Ocimum basilicum* L. cv. Italiano Classico) and tomato (*Solanum lycopersicum* L. cv. Roma V.F.), particularly evident at the foliar level [30]. Our results were consistent with previous findings: 10% FSCG significantly increased *G. longituba* biomass and node number. This improvement is likely due to nutrients released from FSCG, such as nitrogen, phosphorus, iron, and zinc, which enhance soil fertility [14,29,70]. However, plant growth (assessed by biomass and node number) declined at 20% FSCG compared to 10%, showing a dose-dependent response. This inhibition at high FSCG concentration may result from several factors. First, residual phytotoxic compounds like caffeine and polyphenols might accumulate and directly inhibit growth [17,24,27]. Second, caffeine can accelerate plant metabolism, potentially depleting energy reserves [17,18]. Third, high application rates may cause adverse soil changes, such as oxygen depletion or organic acid accumulation [20,29].

Earthworms generally benefit plants through enhanced nutrient cycling [33,71,72]. They improve crop yields in maize, rice, and wheat via nitrogen mineralization [73] and can alleviate stress in contaminated soils [74]. Our data partially support these positive effects, showing that earthworms significantly increased aboveground biomass and node number in *G. longituba*.

Although two-way ANOVA showed no statistically significant FSCG × earthworm interaction for biomass measurements, Tukey HSD test revealed earthworm-mediated modulations. Earthworms reduced the growth stimulation at 10% FSCG but alleviated the inhibition at 20% FSCG. Notably, earthworms significantly changed biomass allocation patterns. They suppressed root investment at 10% FSCG but enhanced it at 20% FSCG. Although direct quantification of soil structural changes was not conducted in this study, the altered root-shoot ratio provides strong evidence that earthworms modified the belowground environment and influenced plant resource allocation. This shift likely involves multiple mechanisms. Earthworm activities like burrowing and casting improve soil structure and accelerate microbial turnover [35–37], thereby influencing nutrient availability across FSCG levels. They may also trigger a trade – off between growth and the production of secondary metabolites in plants [75,76]. Under 10% FSCG, *G. longituba* may prioritize shoot development over root expansion and secondary metabolite synthesis. Under 20% FSCG, they may invest more in roots to acquire resources. These findings highlight the need to optimize FSCG rates and consider soil fauna in agricultural management.

### Responses of phytochemical content

Studies have shown that incorporating FSCG into the soil can trigger a range of physiological changes in vegetables [70,77]. For instance, the addition of FSCG at a concentration of 15% (v/v) significantly increased the levels of essential macronutrients in lettuce (*Lactuca sativa* L. var. *capitata* cv. ‘Four Seasons’), thereby improving its nutritional value and overall quality [77]. This enhancement likely comes from essential elements released by FSCG. These nutrients support photosynthesis, protein synthesis, and energy production. Ronga (2016) [30] found that FSCG enhanced antioxidant capacity in basil and tomato. Antioxidants help plants resist oxidative stress from UV radiation, pollution, and pathogens [78,79]. In our study, plants grown in soil added with 20% exhibited significantly higher levels of total flavonoids, chlorogenic acid, and soluble protein. Flavonoids, known for their antioxidant, anti-inflammatory, and anticancer properties [80,81], and chlorogenic acid, recognized for its antioxidant, antibacterial, and antiviral activities [82,83], both contribute to the medicinal value of the plant. Meanwhile, the elevated levels of soluble protein indicate enhanced plant growth and metabolic activity [84]. These improvements in phytochemical content are likely attributed to the combined effects of improved nutrient availability, shifts in soil microbial communities, and direct bioactive stimulation from FSCG [85,86].

However, earthworms reduced chlorogenic acid content despite promoting growth. This contrast suggests a trade-off between growth and the production of secondary metabolites [50,87]. Several mechanisms may explain this suppression: First, earthworms enhance nutrient mobilization, especially nitrogen [33,73]. This may downregulate the phenylpropanoid pathway, reducing chlorogenic acid production as plants prioritize growth [50,87]. Second, earthworms alter soil microbial community structure [88,89]. This may disadvantage microbes that support chlorogenic acid synthesis while favoring those that boost nitrogen mineralization [85,90]. Third, earthworms can change soil properties like pH [91,92]. This affects nutrient availability and enzyme activity involved in phenolic synthesis [93,94]. These findings have practical implications. FSCG shows potential as a soil amendment to enhance crop quality. However, earthworms’ negative effect on chlorogenic acid highlights the need to understand soil biota-plant interactions. Effective management of soil amendments and fauna could improve crop quality, reduce synthetic inputs, and support sustainable agriculture.

## Conclusion

Our findings demonstrate that FSCG effects on *G. longituba* are concentration-dependent. A 10% FSCG optimally promoted plant growth. In contrast, 20% FSCG maximized the accumulation of total flavonoids, chlorogenic acid, and soluble protein. Earthworm presence stimulated plant growth but reduced chlorogenic acid content. This shows contrasting effects on biomass production and specific secondary metabolite synthesis. Therefore, targeted FSCG application is recommended (e.g., use 10% FSCG for biomass production and 20% FSCG to enhance specific phytochemical constituents). From an agricultural perspective, earthworm management should align with cultivation goals. Earthworms benefit biomass production but may need control when maximizing medicinal compounds like chlorogenic acid. The results support FSCG as a sustainable soil amendment for medicinal plants. Nevertheless, the limitations of this study should be acknowledged: the use of a controlled greenhouse environment may not fully replicate field conditions with natural environmental fluctuations; the phytochemical analysis was restricted to a predefined set of constituents; and the precise mechanisms by which earthworms modulate the soil microbial community, thereby influencing chlorogenic acid biosynthesis, remain unclear. Future research should optimize FSCG concentration and earthworm management for medicinal plants. Studies should consider soil type, climate, and growth stages. Further investigation is needed into the mechanisms through which earthworms affect specific plant metabolites.

## Supporting information

S1 File Supporting information. Table S1. The original data of the effects of fermented spent coffee grounds, earthworm, and their interaction on growth performance of *Glechoma longituba*. Table S2. The original data of the effects of fermented spent coffee grounds, earthworm, and their interaction on content of total flavonoids, chlorogenic acid, soluble sugar, and soluble protein of *Glechoma longituba.* (DOCX)

## References

[1] Marrelli M. Medicinal Plants. Plants (Basel). 2021;10(7):1355. doi: 10.3390/plants10071355 34371558 PMC8309240

[2] Mwangi RW, Macharia JM, Wagara IN, Bence RL. The medicinal properties of Cassia fistula L: A review. Biomedicine & Pharmacotherapy. 2021;144:112240. doi: 10.1016/j.biopha.2021.112240 34601194

[3] Chen J, Zhang X, Zhao J, Ding W, Zhang X, Pan L, et al. Study of the Correlation Between Endophyte Abundances and Metabolite Levels in Different Parts of the Tissue of Cultivated and Wild Arnebia euchroma (Royle) Johnst. Based on Microbiome Analysis and Metabolomics. Molecules. 2025;30(3):734. doi: 10.3390/molecules30030734 39942836 PMC11820562

[4] Rahman IU, Afzal A, Iqbal Z, Hart R, Abd Allah EF, Hashem A, et al. Herbal Teas and Drinks: Folk Medicine of the Manoor Valley, Lesser Himalaya, Pakistan. Plants (Basel). 2019;8(12):581. doi: 10.3390/plants8120581 31817913 PMC6963793

[5] Tang P, Shen T, Wang H, Zhang R, Zhang X, Li X, et al. Challenges and opportunities for improving the druggability of natural product: Why need drug delivery system?. Biomed Pharmacother. 2023;164:114955. doi: 10.1016/j.biopha.2023.114955 37269810

[6] Wu S, Wang C, Bai D, Chen N, Hu J, Zhang J. Perspectives of international multi-center clinical trials on traditional Chinese herbal medicine. Front Pharmacol. 2023;14:1195364. doi: 10.3389/fphar.2023.1195364 37274102 PMC10232835

[7] Hao D-C, Xiao P-G. Genomics and Evolution in Traditional Medicinal Plants: Road to a Healthier Life. Evol Bioinform Online. 2015;11:197–212. doi: 10.4137/EBO.S31326 26461812 PMC4597484

[8] Wang W, Xu J, Fang H, Li Z, Li M. Advances and challenges in medicinal plant breeding. Plant Sci. 2020;298:110573. doi: 10.1016/j.plantsci.2020.110573 32771174

[9] Wang Y, Zhang Y, Cong H, Li C, Wu J, Li L, et al. Cultivable Endophyte Resources in Medicinal Plants and Effects on Hosts. Life (Basel). 2023;13(8):1695. doi: 10.3390/life13081695 37629552 PMC10455732

[10] Parashiva J, Nuthan BR, Rakshith D, Satish S. Endophytic Fungi as a Promising Source of Anticancer L-Asparaginase: A Review. Curr Microbiol. 2023;80(9):282. doi: 10.1007/s00284-023-03392-z 37450223

[11] Gurib-Fakim A. Medicinal plants: traditions of yesterday and drugs of tomorrow. Mol Aspects Med. 2006;27(1):1–93. doi: 10.1016/j.mam.2005.07.008 16105678

[12] Máthé A, Máthé I. Quality assurance of cultivated and gathered medicinal plants. Acta Hortic. 2008;(765):67–76. doi: 10.17660/actahortic.2008.765.8

[13] Angeloni S, Caprioli G, Cespi M, Acquaticci L, Mustafa AM, Santanatoglia A, et al. An innovative formulation to improve spent coffee characteristics as soil fertilizer: Nutrient, chemical characterization and effects on plant germination. Biocatalysis and Agricultural Biotechnology. 2024;61:103394. doi: 10.1016/j.bcab.2024.103394

[14] Cervera-Mata A, Navarro-Alarcón M, Delgado G, Pastoriza S, Montilla-Gómez J, Llopis J, et al. Spent coffee grounds improve the nutritional value in elements of lettuce (Lactuca sativa L.) and are an ecological alternative to inorganic fertilizers. Food Chem. 2019;282:1–8. doi: 10.1016/j.foodchem.2018.12.101 30711092

[15] Horgan FG, Floyd D, Mundaca EA, Crisol-Martínez E. Spent Coffee Grounds Applied as a Top-Dressing or Incorporated into the Soil Can Improve Plant Growth While Reducing Slug Herbivory. Agriculture. 2023;13(2):257. doi: 10.3390/agriculture13020257

[16] Santos C, Fonseca J, Aires A, Coutinho J, Trindade H. Effect of different rates of spent coffee grounds (SCG) on composting process, gaseous emissions and quality of end-product. Waste Manag. 2017;59:37–47. doi: 10.1016/j.wasman.2016.10.020 28340969

[17] Bouhzam I, Cantero R, Balcells M, Margallo M, Aldaco R, Bala A, et al. Environmental and Yield Comparison of Quick Extraction Methods for Caffeine and Chlorogenic Acid from Spent Coffee Grounds. Foods. 2023;12(4):779. doi: 10.3390/foods12040779 36832852 PMC9955646

[18] Murthy PS, Madhava Naidu M. Sustainable management of coffee industry by-products and value addition—A review. Resources, Conservation and Recycling. 2012;66:45–58. doi: 10.1016/j.resconrec.2012.06.005

[19] Ribeiro JP, Vicente ED, Gomes AP, Nunes MI, Alves C, Tarelho LAC. Effect of industrial and domestic ash from biomass combustion, and spent coffee grounds, on soil fertility and plant growth: experiments at field conditions. Environ Sci Pollut Res Int. 2017;24(18):15270–7. doi: 10.1007/s11356-017-9134-y 28500551

[20] Yamane K, Kono M, Fukunaga T, Iwai K, Sekine R, Watanabe Y, et al. Field Evaluation of Coffee Grounds Application for Crop Growth Enhancement, Weed Control, and Soil Improvement. Plant Production Science. 2014;17(1):93–102. doi: 10.1626/pps.17.93

[21] Afriliana A, Hidayat E, Mitoma Y, Masuda T, Harada H. Studies on composting spent coffee grounds by Aspergillus sp and Aspergillus spin aerobic static batch temperature control. J Agric Chem Environ. 2021;10(1):91–112.

[22] Campos-Vega R, Loarca-Piña G, Vergara-Castañeda HA, Oomah BD. Spent coffee grounds: A review on current research and future prospects. Trends in Food Science & Technology. 2015;45(1):24–36. doi: 10.1016/j.tifs.2015.04.012

[23] Kasongo RK, Verdoodt A, Kanyankagote P, Baert G, Ranst EV. Coffee waste as an alternative fertilizer with soil improving properties for sandy soils in humid tropical environments. Soil Use and Management. 2010;27(1):94–102. doi: 10.1111/j.1475-2743.2010.00315.x

[24] Cervera-Mata A, Pastoriza S, Rufián-Henares JÁ, Párraga J, Martín-García JM, Delgado G. Impact of spent coffee grounds as organic amendment on soil fertility and lettuce growth in two Mediterranean agricultural soils. Archives of Agronomy and Soil Science. 2018;64(6):790–804. doi: 10.1080/03650340.2017.1387651

[25] Chilosi G, Aleandri MP, Luccioli E, Stazi SR, Marabottini R, Morales-Rodríguez C, et al. Suppression of soil-borne plant pathogens in growing media amended with espresso spent coffee grounds as a carrier of Trichoderma spp. Scientia Horticulturae. 2020;259:108666. doi: 10.1016/j.scienta.2019.108666

[26] Ciesielczuk T, Rosik-Dulewska C, Poluszyńska J, Miłek D, Szewczyk A, Sławińska I. Acute Toxicity of Experimental Fertilizers Made of Spent Coffee Grounds. Waste Biomass Valor. 2018;9(11):2157–64. doi: 10.1007/s12649-017-9980-3

[27] Cruz R, Baptista P, Cunha S, Pereira JA, Casal S. Carotenoids of lettuce (Lactuca sativa L.) grown on soil enriched with spent coffee grounds. Molecules. 2012;17(2):1535–47. doi: 10.3390/molecules17021535 22314378 PMC6269015

[28] Hardgrove SJ, Livesley SJ. Applying spent coffee grounds directly to urban agriculture soils greatly reduces plant growth. Urban Forestry & Urban Greening. 2016;18:1–8. doi: 10.1016/j.ufug.2016.02.015

[29] Cervera-Mata A, Navarro-Alarcón M, Rufián-Henares JÁ, Pastoriza S, Montilla-Gómez J, Delgado G. Phytotoxicity and chelating capacity of spent coffee grounds: Two contrasting faces in its use as soil organic amendment. Sci Total Environ. 2020;717:137247. doi: 10.1016/j.scitotenv.2020.137247 32092806

[30] Ronga D, Pane C, Zaccardelli M, Pecchioni N. Use of Spent Coffee Ground Compost in Peat-Based Growing Media for the Production of Basil and Tomato Potting Plants. Communications in Soil Science and Plant Analysis. 2016;47(3):356–68. doi: 10.1080/00103624.2015.1122803

[31] Ma L, Song D, Liu M, Li Y, Li Y. Effects of earthworm activities on soil nutrients and microbial diversity under different tillage measures. Soil and Tillage Research. 2022;222:105441. doi: 10.1016/j.still.2022.105441

[32] Mudrák O, Frouz J. Earthworms increase plant biomass more in soil with no earthworm legacy than in earthworm‐mediated soil, and favour late successional species in competition. Functional Ecology. 2017;32(3):626–35. doi: 10.1111/1365-2435.12999

[33] van Groenigen JW, Lubbers IM, Vos HMJ, Brown GG, De Deyn GB, van Groenigen KJ. Earthworms increase plant production: a meta-analysis. Sci Rep. 2014;4:6365. doi: 10.1038/srep06365 25219785 PMC5376159

[34] Wang D, Song F, Zhou Y, Zhong T, Zhang Y, Deng Q, et al. Effects of alkaline salt stress on growth, physiological properties and medicinal components of clonal Glechoma longituba (Nakai) Kupr. BMC Plant Biol. 2024;24(1):965. doi: 10.1186/s12870-024-05668-3 39402458 PMC11475845

[35] Don A, Steinberg B, Schöning I, Pritsch K, Joschko M, Gleixner G, et al. Organic carbon sequestration in earthworm burrows. Soil Biology and Biochemistry. 2008;40(7):1803–12. doi: 10.1016/j.soilbio.2008.03.003

[36] Römbke J, Jänsch S, Didden W. The use of earthworms in ecological soil classification and assessment concepts. Ecotoxicol Environ Saf. 2005;62(2):249–65. doi: 10.1016/j.ecoenv.2005.03.027 15922446

[37] Shuster WD, Subler S, McCoy EL. Deep-burrowing earthworm additions changed the distribution of soil organic carbon in a chisel-tilled soil. Soil Biology and Biochemistry. 2001;33(7–8):983–96. doi: 10.1016/s0038-0717(01)00002-5

[38] Wu Y, Deng S, Hao P, Tang H, Xu Y, Zhang Y, et al. Roxarsone reduces earthworm-mediated nutrient cycling by suppressing aggregate formation and enzymic activity in soil with manure application. Environ Pollut. 2024;360:124777. doi: 10.1016/j.envpol.2024.124777 39173866

[39] Huang C, Wang L, Wu W-M, Capowiez Y, Qiao Y, Hou D. When plastisphere and drilosphere meet: Earthworms facilitate microbiome and nutrient turnover to accelerate biodegradation of agricultural plastic films. Environ Int. 2025;196:109309. doi: 10.1016/j.envint.2025.109309 39904096

[40] Araujo Y, Luizo FJ, Barros E. Effect of earthworm addition on soil nitrogen availability, microbial biomass and litter decomposition in mesocosms. Biology and Fertility of Soils. 2004;39(3):146–52. doi: 10.1007/s00374-003-0696-0

[41] Azeem A, Mai W, Gul B, Rasheed A. Influence of Soil Amendment Application on Growth and Yield of Hedysarum scoparium Fisch. et Mey and Avena sativa L. Under Saline Conditions in Dry-Land Regions. Plants (Basel). 2025;14(6):855. doi: 10.3390/plants14060855 40265764 PMC11945427

[42] Gómez-Brandón M, Lores M, Domínguez J. Species-specific effects of epigeic earthworms on microbial community structure during first stages of decomposition of organic matter. PLoS One. 2012;7(2):e31895. doi: 10.1371/journal.pone.0031895 22363763 PMC3283695

[43] Hafez EM, Omara AED, Alhumaydhi FA, El-Esawi MA. Minimizing hazard impacts of soil salinity and water stress on wheat plants by soil application of vermicompost and biochar. Physiol Plant. 2020;172(2):587–602. doi: 10.1111/ppl.13261 33159342

[44] Tikoria R, Kaur A, Ohri P. Physiological, biochemical and structural changes in tomato plants by vermicompost application in different exposure periods under glass house conditions. Plant Physiol Biochem. 2023;197:107656. doi: 10.1016/j.plaphy.2023.107656 37001305

[45] Yusof Z, Ramasamy S, Mahmood NZ, Yaacob JS. Vermicompost Supplementation Improves the Stability of Bioactive Anthocyanin and Phenolic Compounds in Clinacanthus nutans Lindau. Molecules. 2018;23(6):1345. doi: 10.3390/molecules23061345 29867000 PMC6100045

[46] Mendes LA, Domínguez J. Spent coffee grounds as a suitable alternative to standard soil in ecotoxicological tests. Environ Sci Pollut Res Int. 2024;31(11):16725–34. doi: 10.1007/s11356-024-32297-y 38326678 PMC10894160

[47] Cui J, Jiang J, Chang E, Zhang F, Guo L, Fang D, et al. Underlying reasons and factors associated with changes in earthworm activities in response to biochar amendment: a review. Biochar. 2023;5(1). doi: 10.1007/s42773-023-00287-x

[48] Hou S, Wang J, Dai J, Boussafir M, Zhang C. Combined effects of earthworms and biochar on PAHs-contaminated soil remediation: A review. Soil Ecol Lett. 2022;5(2). doi: 10.1007/s42832-022-0158-y

[49] Li Y, Qu N, Li S, Zhou H, Yue M. Ecological mechanisms of microbial assembly in clonal plant Glechoma longituba: from soil to endosphere. Appl Environ Microbiol. 2025;91(6):e0033625. doi: 10.1128/aem.00336-25 40353652 PMC12175508

[50] Zhao B-N, Wang X-G, Zhang R, He X-G, Xie Z-Y, Yao X-Q, et al. The effects of clonal integration and earthworms on the growth, active constituent accumulation in Glechoma longituba, and the soil microbial community in its root zone. Front Plant Sci. 2025;16:1596905. doi: 10.3389/fpls.2025.1596905 41031308 PMC12477172

[51] Zhang R, Chen Z-H, Li Y-M, Wang N, Cui W-T, Zhao B-N, et al. Effects of clonal integration and nutrient availability on the growth of Glechoma longituba under heterogenous light conditions. Front Plant Sci. 2023;14. doi: 10.3389/fpls.2023.1182068 PMC10465176 37649995

[52] Chu Y, Yu F, Dong M. Clonal Plasticity in Response to Reciprocal Patchiness of Light and Nutrients in the Stoloniferous Herb Glechoma longituba L. JIPB. 2006;48(4):400–8. doi: 10.1111/j.1744-7909.2006.00237.x

[53] Xiong H-Q. Soil nutrient patchiness affects nutrient use efficiency, though not photosynthesis and growth of parental Glechoma longituba ramets: both patch contrast and direction matter. Journal of Plant Ecology. 2010;3(2):131–7. doi: 10.1093/jpe/rtq010

[54] Quan J, Latzel V, Tie D, Zhang Y, Münzbergová Z, Chai Y, et al. Ultraviolet B Radiation Triggers DNA Methylation Change and Affects Foraging Behavior of the Clonal Plant Glechoma longituba. Front Plant Sci. 2021;12:633982. doi: 10.3389/fpls.2021.633982 33719308 PMC7952652

[55] Xing L, Quan J, Zhang S, Liu X, Bai H, Yue M. Changes induced by parental neighboring touch in the clonal plant Glechoma longituba depend on the light environment. Front Plant Sci. 2024;15:1358924. doi: 10.3389/fpls.2024.1358924 38831907 PMC11146198

[56] Grabowska K, Amanowicz K, Paśko P, Podolak I, Galanty A. Optimization of the Extraction Procedure for the Phenolic-Rich Glechoma hederacea L. Herb and Evaluation of Its Cytotoxic and Antioxidant Potential. Plants (Basel). 2022;11(17):2217. doi: 10.3390/plants11172217 36079600 PMC9460379

[57] Jin L, Liu L, Guo Q, Wang L, Zou J. Variation in bioactive compounds of Glechoma longituba and its influential factors: Implication for advanced cultivation strategies. Scientia Horticulturae. 2019;244:182–92. doi: 10.1016/j.scienta.2018.09.047

[58] Jin L, Liu L, Guo Q. Phosphorus and iron in soil play dominating roles in regulating bioactive compounds of Glechoma longituba (Nakai) Kupr. Scientia Horticulturae. 2019;256:108534. doi: 10.1016/j.scienta.2019.05.061

[59] Lowe C, Butt K. Growth of hatchling earthworms in the presence of adults: interactions in laboratory culture. Biology and Fertility of Soils. 2002;35(3):204–9. doi: 10.1007/s00374-002-0471-7

[60] Meng L-L, Srivastava AK, Kuča K, Wu Q-S. Earthworm (Pheretima guillelmi)-mycorrhizal fungi (Funneliformis mosseae) association mediates rhizosphere responses in white clover. Applied Soil Ecology. 2022;172:104371. doi: 10.1016/j.apsoil.2021.104371

[61] Chang X, Fu F, Sun Y, Zhao L, Li X, Li Y. Coupling multifactor dominated the biochemical response and the alterations of intestinal microflora of earthworm Pheretima guillelmi due to typical herbicides. Environ Sci Pollut Res Int. 2023;30(41):94126–37. doi: 10.1007/s11356-023-29032-4 37526832

[62] Si C, Xue W, Guo Z-W, Zhang J-F, Hong M-M, Wang Y-Y, et al. Soil heterogeneity and earthworms independently promote growth of two bamboo species. Ecological Indicators. 2021;130:108068. doi: 10.1016/j.ecolind.2021.108068

[63] Zheng X, Fan X, Zhang H, Li S, Wang J, Zhang J, et al. Effects of *Pheretima guillelmi* cultivation time on microbial community diversity and characteristics of carbon metabolism in vegetable Soil. J Agric Resour Environ. 2015;32:596. doi: 10.13254/j.jare.2015.0139

[64] Meng L-L, Srivastava AK, Kuča K, Wu Q-S. Earthworm (Pheretima guillelmi)-mycorrhizal fungi (Funneliformis mosseae) association mediates rhizosphere responses in white clover. Applied Soil Ecology. 2022;172:104371. doi: 10.1016/j.apsoil.2021.104371

[65] Tao J, Griffiths B, Zhang S, Chen X, Liu M, Hu F, et al. Effects of earthworms on soil enzyme activity in an organic residue amended rice–wheat rotation agro-ecosystem. Applied Soil Ecology. 2009;42(3):221–6. doi: 10.1016/j.apsoil.2009.04.003

[66] Liu J, Li C, Ding G, Quan W. Artificial Intelligence Assisted Ultrasonic Extraction of Total Flavonoids from Rosa sterilis. Molecules. 2021;26(13):3835. doi: 10.3390/molecules26133835 34201870 PMC8270336

[67] Oteef MDY. Comparison of Different Extraction Techniques and Conditions for Optimizing an HPLC-DAD Method for the Routine Determination of the Content of Chlorogenic Acids in Green Coffee Beans. Separations. 2022;9(12):396. doi: 10.3390/separations9120396

[68] Gao JF. Experimental guidance of plant physiology. 2006;142:144.

[69] Kumar A, Thakur MK, Hart P, Thakur VK. Sustainable Valorization of Spent Coffee Grounds: A Green Chemistry Approach to Soil Amendment and Environmental Monitoring. ACS Sustain Resour Manag. 2025;2(9):1630–42. doi: 10.1021/acssusresmgt.5c00083 41031148 PMC12478863

[70] Cruz R, Morais S, Mendes E, Pereira JA, Baptista P, Casal S. Improvement of vegetables elemental quality by espresso coffee residues. Food Chem. 2014;148:294–9. doi: 10.1016/j.foodchem.2013.10.059 24262560

[71] McCormick MK, Parker KL, Szlavecz K, Whigham DF. Native and exotic earthworms affect orchid seed loss. AoB Plants. 2013;5(0):plt018–plt018. doi: 10.1093/aobpla/plt018

[72] Adomako MO, Xue W, Roiloa S, Zhang Q, Du D-L, Yu F-H. Earthworms Modulate Impacts of Soil Heterogeneity on Plant Growth at Different Spatial Scales. Front Plant Sci. 2021;12:735495. doi: 10.3389/fpls.2021.735495 35003149 PMC8732864

[73] Xiao Z, Wang X, Koricheva J, Kergunteuil A, Le Bayon R, Liu M, et al. Earthworms affect plant growth and resistance against herbivores: A meta‐analysis. Functional Ecology. 2018;32(1):150–60. doi: 10.1111/1365-2435.12969

[74] Li Y, Wang J, Shao M, Jia H. Earthworm activity effectively mitigated the negative impact of microplastics on maize growth. J Hazard Mater. 2023;459:132121. doi: 10.1016/j.jhazmat.2023.132121 37499490

[75] Hiruma K. Roles of Plant-Derived Secondary Metabolites during Interactions with Pathogenic and Beneficial Microbes under Conditions of Environmental Stress. Microorganisms. 2019;7(9):362. doi: 10.3390/microorganisms7090362 31540419 PMC6780457

[76] Jian S-F, Huang X-J, Yang X-N, Zhong C, Miao J-H. Sulfur Regulates the Trade-Off Between Growth and Andrographolide Accumulation via Nitrogen Metabolism in Andrographis paniculata. Front Plant Sci. 2021;12:687954. doi: 10.3389/fpls.2021.687954 34335655 PMC8317024

[77] Cruz R, Gomes T, Ferreira A, Mendes E, Baptista P, Cunha S, et al. Antioxidant activity and bioactive compounds of lettuce improved by espresso coffee residues. Food Chem. 2014;145:95–101. doi: 10.1016/j.foodchem.2013.08.038 24128454

[78] Khan MIR, Khan NA. Reactive Oxygen Species and Antioxidant Systems in Plants: Role and Regulation under Abiotic Stress. Springer Singapore. 2017. doi: 10.1007/978-981-10-5254-5

[79] Rao MJ, Duan M, Zhou C, Jiao J, Cheng P, Yang L, et al. Antioxidant Defense System in Plants: Reactive Oxygen Species Production, Signaling, and Scavenging During Abiotic Stress-Induced Oxidative Damage. Horticulturae. 2025;11(5):477. doi: 10.3390/horticulturae11050477

[80] Gorniak RKJ. Comprehensive review of antimicrobial activities of plant flavonoids. Phytochem Rev: Proceedings of the Phytochemical Society of Europe. 2019;18(1).

[81] Vaibhavi V, Nikita K, Pradnya T, Pratiksha S. Biological activity of flavonoids. Int J Pharm Sci. 2025;3(6):1672–8. doi: 10.5281/zenodo.15619295

[82] Huang J, Xie M, He L, Song X, Cao T. Chlorogenic acid: a review on its mechanisms of anti-inflammation, disease treatment, and related delivery systems. Front Pharmacol. 2023;14:1218015. doi: 10.3389/fphar.2023.1218015 37781708 PMC10534970

[83] Miao M, Xiang L. Pharmacological action and potential targets of chlorogenic acid. Adv Pharmacol. 2020;87:71–88. doi: 10.1016/bs.apha.2019.12.002 32089239

[84] Ren H-Y, Qian W-Z, Yi L, Ye Y-L, Gu T, Gao S, et al. Nutrient Composition and Antioxidant Activity of Cercis chinensis Flower in Response to Different Development Stages. Horticulturae. 2023;9(9):961. doi: 10.3390/horticulturae9090961

[85] Pérez-Burillo S, Cervera-Mata A, Fernández-Arteaga A, Pastoriza S, Rufián-Henares JÁ, Delgado G. Why Should We Be Concerned with the Use of Spent Coffee Grounds as an Organic Amendment of Soils? A Narrative Review. Agronomy. 2022;12(11):2771. doi: 10.3390/agronomy12112771

[86] Vela-Cano M, Cervera-Mata A, Purswani J, Pozo C, Delgado G, González-López J. Bacterial community structure of two Mediterranean agricultural soils amended with spent coffee grounds. Applied Soil Ecology. 2019;137:12–20. doi: 10.1016/j.apsoil.2019.01.006

[87] Hodson ME, Brailey-Crane P, Burn WL, Harper AL, Hartley SE, Helgason T, et al. Enhanced plant growth in the presence of earthworms correlates with changes in soil microbiota but not nutrient availability. Geoderma. 2023;433:116426. doi: 10.1016/j.geoderma.2023.116426

[88] Ferlian O, Goldmann K, Bonkowski M, Dumack K, Wubet T, Eisenhauer N. Invasive earthworms shift soil microbial community structure in northern North American forest ecosystems. iScience. 2024;27(2):108889. doi: 10.1016/j.isci.2024.108889 38322986 PMC10844042

[89] Gong X, Wang D, Xu M, Du Y, Chen X, Hu F, et al. Earthworm ecotype diversity mitigates resource limitations of microbial community in arable soils. Soil Biology and Biochemistry. 2023;182:109040. doi: 10.1016/j.soilbio.2023.109040

[90] Kang Y, Wu H, Guan Q, Zhang Z, Wang W. Earthworms and warming alter methane uptake and methane-cycling microbial community in meadow soil. Soil Ecol Lett. 2024;6(4). doi: 10.1007/s42832-024-0255-1

[91] García-Montero LG, Valverde-Asenjo I, Grande-Ortíz MA, Menta C, Hernando I. Impact of earthworm casts on soil pH and calcium carbonate in black truffle burns. Agroforest Syst. 2013;87(4):815–26. doi: 10.1007/s10457-013-9598-9

[92] Rayela RNE, Fetiza APS, Estorico GC. The Impact of Soil pH on Earthworm Diversity and Abundance: A Systematic Review of Soil Acidity and its Effects on Vermicommunities. International Journal of Innovative Science and Research Technology (IJISRT). 2025;488–94. doi: 10.38124/ijisrt/25apr031

[93] Xin P, Zhang Y, Jiang N, Chen Z, Chen L. Neutral soil pH conditions favor the inhibition of phenol on hydrolase activities and soil organic carbon mineralization. European Journal of Soil Biology. 2024;121:103621. doi: 10.1016/j.ejsobi.2024.103621

[94] Wang S, Zhang X, Wang Y, Wu J, Lee Y-W, Xu J, et al. NaCl Stress Stimulates Phenolics Biosynthesis and Antioxidant System Enhancement of Quinoa Germinated after Magnetic Field Pretreatment. Foods. 2024;13(20):3278. doi: 10.3390/foods13203278 39456340 PMC11507989

