# Supplementary material for: Concentration-dependent effects of fermented spent coffee grounds and contrasting effects of earthworms on growth and phytochemicals in medicinal plant Glechoma longituba
Source: PLoS One. 2025 Dec 17;20(12):e0339185. doi: 10.1371/journal.pone.0339185 (PMC12711013; doi:10.1371/journal.pone.0339185)
Supplement: S1 File — (DOCX) [file pone.0339185.s001.docx]

Supporting Information

Table S1. The original data of the effects of fermented spent coffee grounds, earthworm, and their interaction on growth performance of *Glechoma longituba*.

| NO. | FSCG tretment | Earthworm treatment | node number | shoot mass (g) | root mass (g) | total mass (g) | shoot- root ratio | |
| --- | --- | --- | --- | --- | --- | --- | --- | --- |
| 0CNO① | 1 | 1 | 26 | 1.151 | 0.156 | 1.307 | | 0.135534318 |
| 0CNO③ | 1 | 1 | 43 | 1.271 | 0.218 | 1.489 | | 0.171518489 |
| 0CNO⑤ | 1 | 1 | 54 | 2.579 | 0.287 | 2.866 | | 0.111283443 |
| 0CEW① | 1 | 2 | 33 | 1.092 | 0.252 | 1.344 | | 0.230769231 |
| 0CEW② | 1 | 2 | 33 | 1.331 | 0.137 | 1.468 | | 0.102930128 |
| 0CEW③ | 1 | 2 | 54 | 2.408 | 0.236 | 2.644 | | 0.098006645 |
| 0CEW④ | 1 | 2 | 74 | 4.308 | 0.477 | 4.785 | | 0.110724234 |
| 0CEW⑤ | 1 | 2 | 76 | 5.634 | 0.699 | 6.333 | | 0.124068158 |
| 10%NO① | 2 | 1 | 55 | 5.728 | 0.863 | 6.591 | | 0.150663408 |
| 10%NO② | 2 | 1 | 73 | 2.796 | 0.26 | 3.056 | | 0.092989986 |
| 10%NO③ | 2 | 1 | 83 | 5.54 | 0.978 | 6.518 | | 0.176534296 |
| 10%NO④ | 2 | 1 | 67 | 5.21 | 0.569 | 5.779 | | 0.109213052 |
| 10%NO⑤ | 2 | 1 | 57 | 5.673 | 0.759 | 6.432 | | 0.133791645 |
| 10%EW① | 2 | 2 | 79 | 5.348 | 0.568 | 5.916 | | 0.106207928 |
| 10%EW② | 2 | 2 | 74 | 5.574 | 0.445 | 6.019 | | 0.079834948 |
| 10%EW③ | 2 | 2 | 74 | 5.434 | 0.479 | 5.913 | | 0.088148693 |
| 10%EW④ | 2 | 2 | 82 | 5.463 | 0.779 | 6.242 | | 0.142595643 |
| 10%EW⑤ | 2 | 2 | 71 | 4.189 | 0.349 | 4.538 | | 0.08331344 |
| 20%NO① | 3 | 1 | 43 | 1.662 | 0.247 | 1.909 | | 0.148616125 |
| 20%NO② | 3 | 1 | 44 | 2.014 | 0.264 | 2.278 | | 0.131082423 |
| 20%NO③ | 3 | 1 | 41 | 2.482 | 0.371 | 2.853 | | 0.149476229 |
| 20%NO④ | 3 | 1 | 51 | 2.008 | 0.209 | 2.217 | | 0.104083665 |
| 20%NO⑤ | 3 | 1 | 53 | 2.464 | 0.462 | 2.926 | | 0.187500000 |
| 20%EW① | 3 | 2 | 80 | 3.162 | 0.523 | 3.685 | | 0.165401645 |
| 20%EW② | 3 | 2 | 49 | 3.283 | 0.643 | 3.926 | | 0.195857447 |
| 20%EW③ | 3 | 2 | 76 | 3.634 | 0.525 | 4.159 | | 0.144468905 |
| 20%EW④ | 3 | 2 | 68 | 3.122 | 0.446 | 3.568 | | 0.142857143 |
| 20%EW⑤ | 3 | 2 | 64 | 3.159 | 0.428 | 3.587 | | 0.135485913 |

Table S2. The original data of the effects of fermented spent coffee grounds, earthworm, and their interaction on content of total flavonoids, chlorogenic acid, soluble sugar, and soluble protein of *Glechoma longituba*.

| NO. | FSCG tretment | Earthworm treatment | Total flavonoids（mg/g） | Chlorogenic acid（mg/g） | Soluble sugar（mg/g） | Soluble protein（mg/g） |
| --- | --- | --- | --- | --- | --- | --- |
| 0CNO① | 1 | 1 | 22.69038414 | 12.31199494 | 78.13846154 | 221.7816771 |
| 0CNO③ | 1 | 1 | 15.4959251 | 10.98823348 | 87.91864199 | 198.0655625 |
| 0CNO⑤ | 1 | 1 | 10.12750484 | 10.47294007 | 116.2512463 | 201.8098718 |
| 0CEW① | 1 | 2 | 11.04484206 | 9.192436428 | 121.4422122 | 221.560117 |
| 0CEW② | 1 | 2 | 16.81122546 | 9.825003173 | 87.67692308 | 240.9434669 |
| 0CEW③ | 1 | 2 | 15.40396116 | 9.201528847 | 74.44615385 | 210.8152509 |
| 0CEW④ | 1 | 2 | 13.67905484 | 9.747181366 | 103.3692308 | 211.8626674 |
| 0CEW⑤ | 1 | 2 | 11.51129529 | 8.717251891 | 91.03964556 | 239.3832801 |
| 10%NO① | 2 | 1 | 15.52682426 | 10.52509615 | 124.227318 | 221.1183221 |
| 10%NO② | 2 | 1 | 9.704491584 | 9.664017341 | 76.6791187 | 236.7296951 |
| 10%NO③ | 2 | 1 | 11.49984127 | 8.844187921 | 118.0455727 | 243.2287441 |
| 10%NO④ | 2 | 1 | 13.69263881 | 11.35680765 | 91.31661203 | 206.408484 |
| 10%NO⑤ | 2 | 1 | 16.36765751 | 10.52509615 | 121.6269841 | 253.162986 |
| 10%EW① | 2 | 2 | 14.14981774 | 10.30412767 | 76.22169703 | 260.1927954 |
| 10%EW② | 2 | 2 | 14.16389716 | 11.27846142 | 116.7538462 | 197.9629953 |
| 10%EW③ | 2 | 2 | 16.72808389 | 9.445599106 | 109.5695971 | 255.602351 |
| 10%EW④ | 2 | 2 | 12.37554592 | 9.370930339 | 91.55610093 | 228.3021517 |
| 10%EW⑤ | 2 | 2 | 11.95574685 | 11.23357202 | 84.55421318 | 237.5724495 |
| 20%NO① | 3 | 1 | 24.7217258 | 14.52665831 | 118.5043585 | 307.1364957 |
| 20%NO② | 3 | 1 | 14.60790647 | 10.36582904 | 111.817279 | 244.5342531 |
| 20%NO③ | 3 | 1 | 22.69038414 | 14.45445822 | 95.41214253 | 328.3075499 |
| 20%NO④ | 3 | 1 | 20.90724982 | 12.44221085 | 122.449292 | 287.5253142 |
| 20%NO⑤ | 3 | 1 | 23.41818774 | 12.50417406 | 116.4005806 | 297.6069683 |
| 20%EW① | 3 | 2 | 17.20349076 | 10.34517997 | 96.65718896 | 283.6260481 |
| 20%EW② | 3 | 2 | 14.6370349 | 11.74940159 | 100.5056696 | 256.1880032 |
| 20%EW③ | 3 | 2 | 16.74464635 | 11.40196394 | 89.43364328 | 282.2384297 |
| 20%EW④ | 3 | 2 | 15.90827616 | 10.99911292 | 111.4116653 | 268.7543602 |
| 20%EW⑤ | 3 | 2 | 16.41661361 | 11.22240545 | 89.86885497 | 223.4187932 |
